# Supplementary material for: LLM-powered socially assistive robot-delivered cognitive behavioral therapy exercises: an exploratory study with university students
Source: Front Robot AI. 2026 Sep 9;13:1802622. doi: 10.3389/frobt.2026.1802622 (PMC13597320; doi:10.3389/frobt.2026.1802622)
Supplement: Supplementary file 1 [file Supplementaryfile1.pdf]

Supplementary Material

0.1 Race/Ethnic Group Distribution

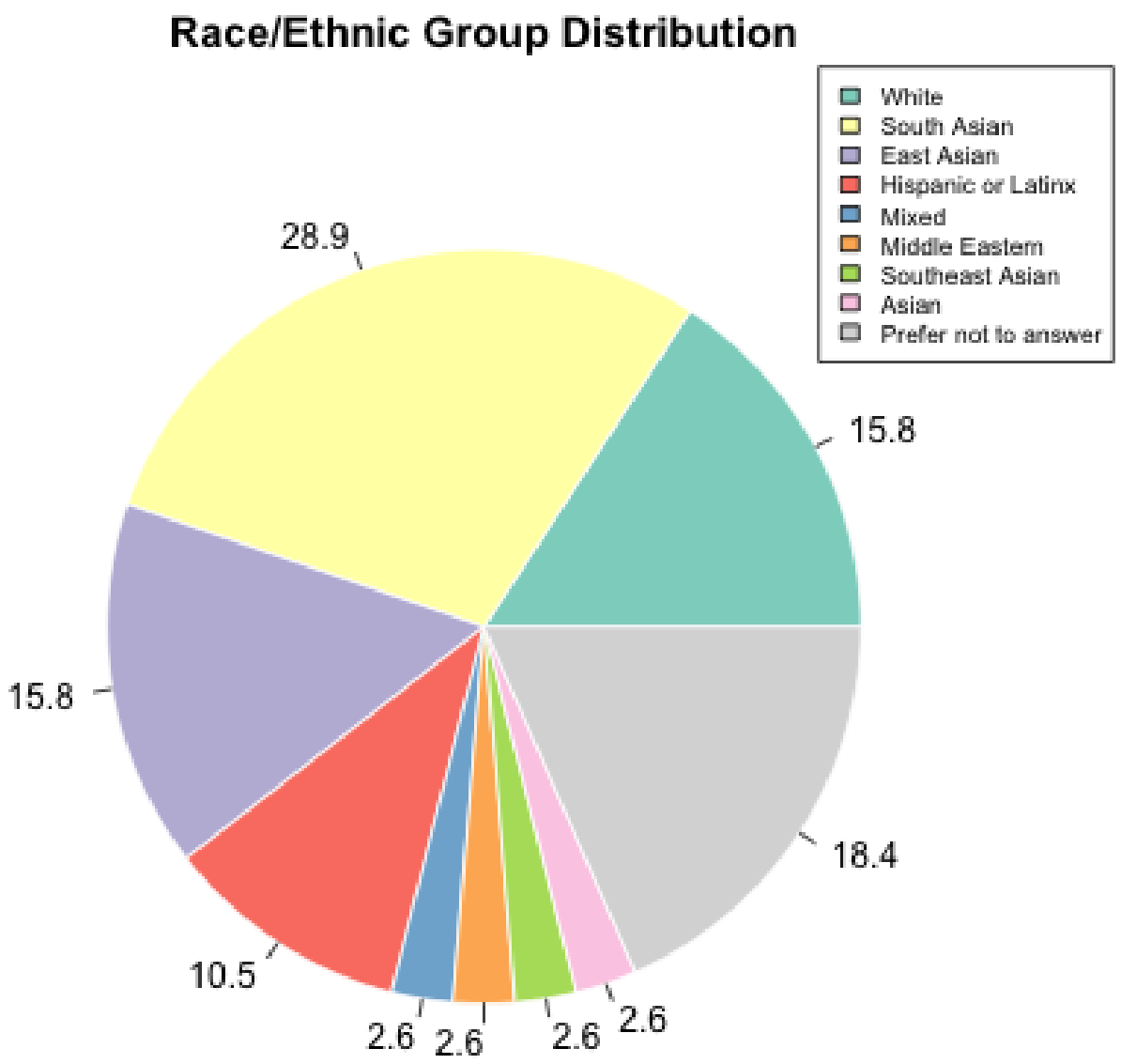

Figure S1. Race/Ethnic Group Distribution Figure

0.2 Blossom Robot Design

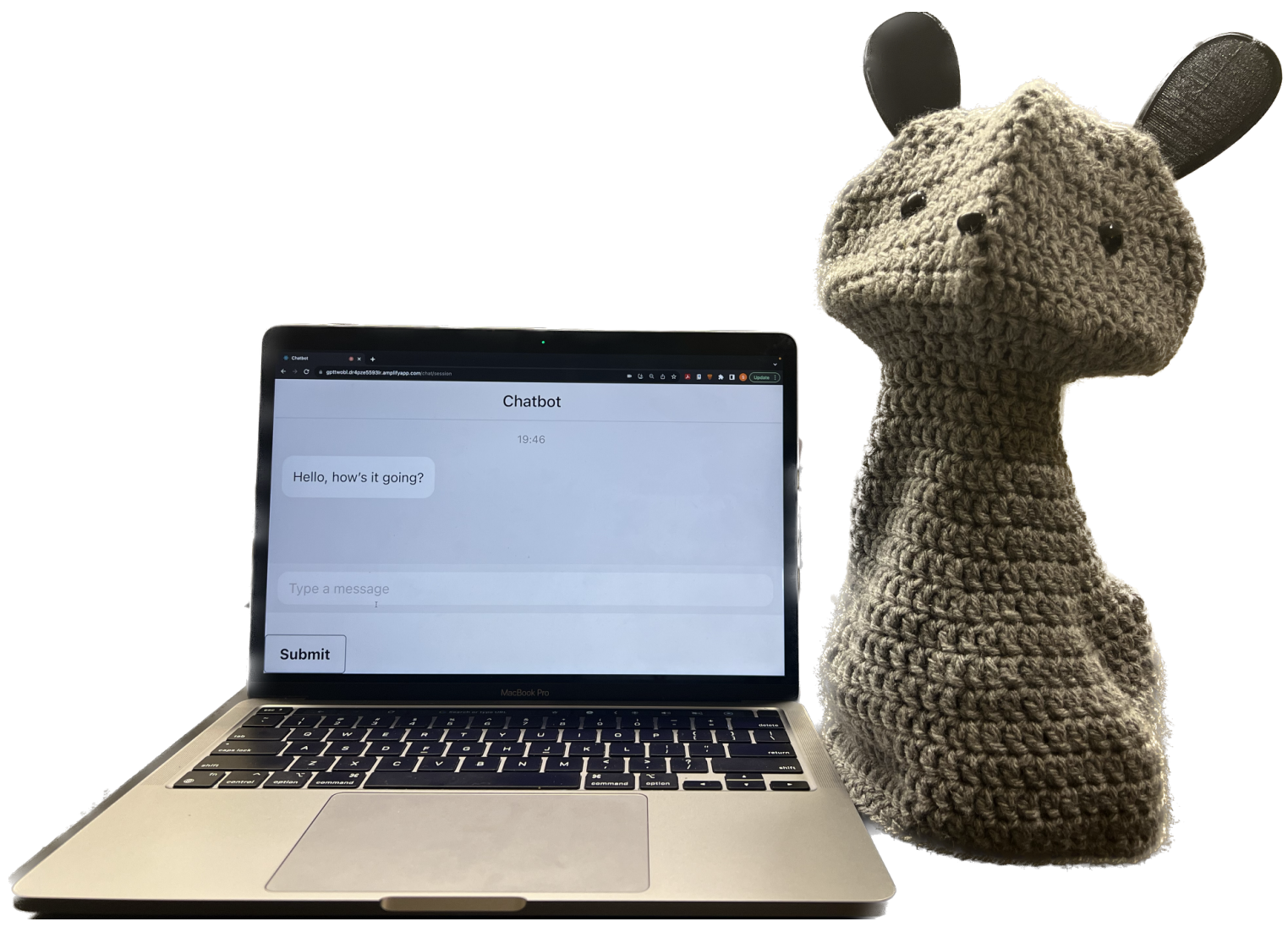

Figure S2. Blossom Robot Design

0.3 GPT-3.5 Parameters

The input parameters for OpenAI's chat completion API were:
The transcript being sent includes all of the previous messages and the user's latest message.

model	gpt-3.5-turbo
messages	<transcript>
stop	Patient
temperature	1
frequency_penalty	2
presence_penalty	2
n	2
max_tokens	150

Table S1. Parameters for OpenAI's completion API

For moderator, the input parameters were same as above, except the temperature was increased to 2 and the values for n and max_tokens were not sent.

0.4 Libraries used for Data Analysis

Python libraries and their versions: R packages:

pandas	2.0.2
matplotlib	3.7.1
scipy	1.10.1
pingouin	0.5.3
numpy	1.24.3

Table S2. Python libraries

readxl	1.4.2
dplyr	1.1.2
tidyr	1.3.0
outliers	0.15
rstatix	0.7.2
ggpubr	0.6.0
car	3.1-2
lmtest	0.9-40
DescTools	0.99.49
ggplot2	3.4.2
ggcorrplot	0.1.4
MASS	7.3-60
afex	1.3-0

Table S3. R packages

1 COGNITIVE RESTRUCTURING WORKSHEET

Socratic Questions

Thoughts are like a running dialogue in your brain. They come and go fast. So fast, in fact, that we rarely have the time to question them. Because our thoughts determine how we feel, and how we act, it's important to challenge any thoughts that cause us harm.

Spend a moment thinking about each of the following questions, and record thorough responses. Elaborate, and explain "why" or "why not" in your responses.

Thought to be questioned:

What is the evidence for this thought? Against it?

Am I basing this thought on facts, or on feelings?

Is this thought black and white, when reality is more complicated?

Could I be misinterpreting the evidence? Am I making any assumptions?

© 2017 Therapist Aid LLC 1 Provided by **TherapistAid.com**

2 COPING STRATEGIES WORKSHEET

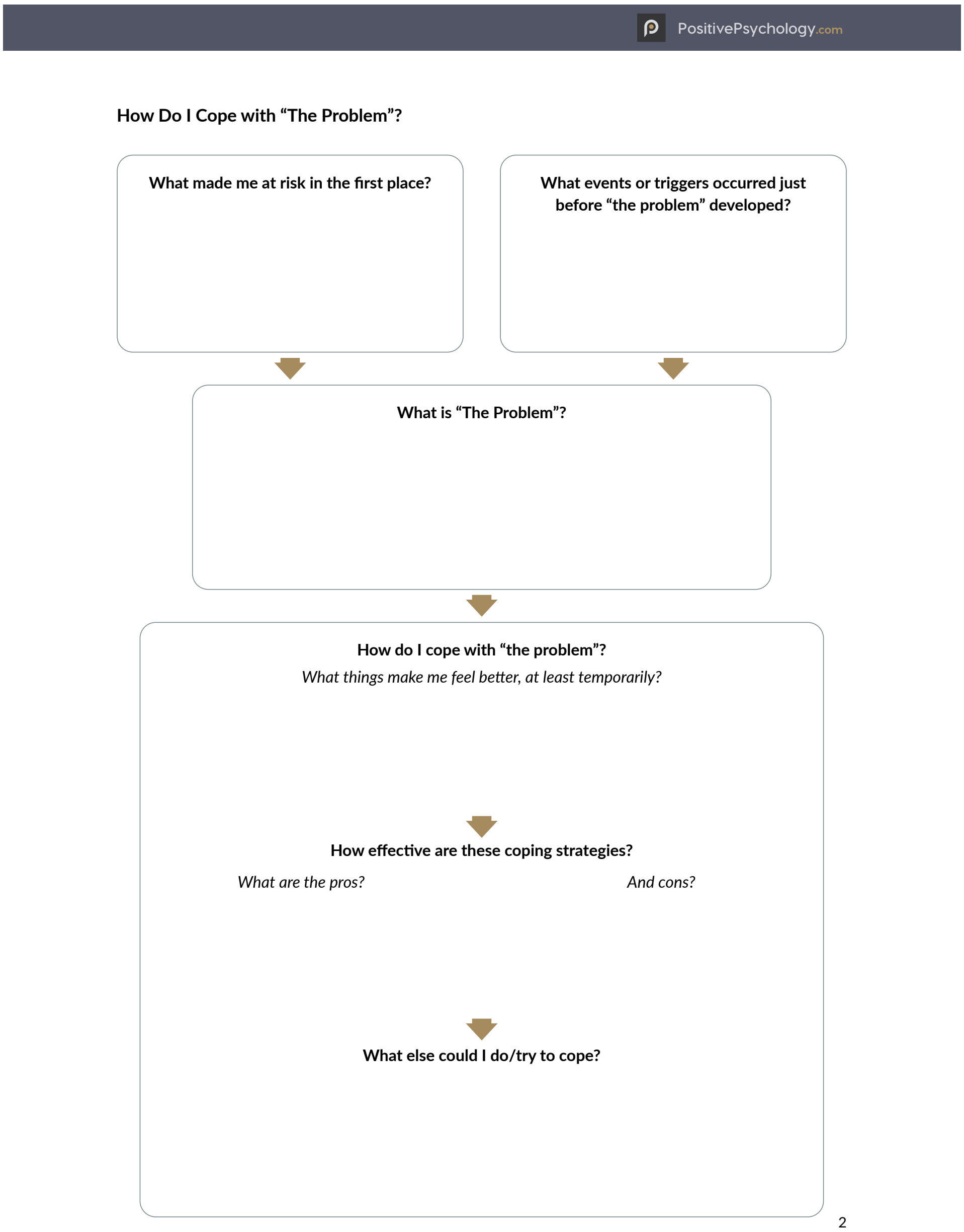
PositivePsychology.com

How Do I Cope with "The Problem"?

What made me at risk in the first place?

What events or triggers occurred just before "the problem" developed?

What is "The Problem"?

How do I cope with "the problem"?
What things make me feel better, at least temporarily?

How effective are these coping strategies?
What are the pros? *And cons?*

What else could I do/try to cope?

2

3 EXTENDED THEMATIC ANALYSIS

3.1 Augmenting Therapy: The Potential Role of SAR CBT Companions in Therapy Practice

Worksheet

Participants in the worksheet conditions emphasized the importance of anonymity in completing the worksheets. Some described feeling uncomfortable opening up to other people, so they were more comfortable writing about their thoughts by themselves. Similarly, they recalled being able to share their

thoughts more freely knowing that no one was listening to them and they were not being judged for the things they wrote: P36 appreciated being able to *" share without judgement [...] knowing it was confidential but also not having someone there expecting my answers."* One participant suggested that items written down in private are more truthful than when said to a person. A few participants noted some drawbacks to filling out a worksheet as an individual, stating that writing their feelings in a worksheet felt one-sided. P36 expressed a desire to be understood that could not be fulfilled by a worksheet *"I'd prefer to talk to a real person though because..[this] technology has never experienced real life."* One participant wanted to know how many other people are dealing with the same problems as them.

Chatbot

One theme that emerged from the chatbot condition is convenience. Participants appreciated that they could use the platform anytime they needed, making the platform good for immediate help. P17 stated, *"[the chatbot] allows you to talk about immediate stress. Couldn't do [that] with a therapist."*, and P16 observed that *"the chatbot aspect makes it convenient. You can pull it up whenever you need it. Since there wasn't a set time, it was convenient for me."* They shared that the exercises fit into a busy schedule *"really easy, because it would only take like 10 mins to check in, especially on a day which was bad"* (P22).

Many participants in the chatbot condition shared that the chatbot was good for completing at-home exercises and addressing minor concerns, but emphasized that it was not a replacement for therapy with a human being: *"For people who aren't in therapy for bigger things, lower level issues, it makes sense that this therapy could be accessible and administered through chatbot."* (P22). Two participants stated that they preferred interacting with a human therapist and had no need for a chatbot.

Like participants in the worksheet condition, a few shared that they felt no hesitation to write about their thoughts because they weren't interacting with a human who could judge them. P26 described the chatbot as *"'more objective than a human."*

Blossom

A few participants shared that Blossom could be helpful in conjunction with a human therapist, but was not an alternative to CBT with a person. They appreciated that the robot adjusted its personality and conversation style to match theirs in a way that humans typically don't, also noting that they felt more comfortable using casual language and punctuation (like texting) with Blossom than they would with a therapist. Additionally, one participant appreciated that the robot could be used without health insurance.

Participants made several comments about the convenience of the robot-guided CBT homework exercises. They spoke positively about being able to complete the exercises at home any time they wanted, without needing to make an appointment. P14 commented *"Having access to a pseudo-therapist was nice, someone I could talk to whenever I want was definitely a highlight'*. P8 compared it to going to see an in-person therapist, stating, *"[in-person] therapy takes a lot of effort (schedule, pay, go to place). It takes a lot of time, so Blossom offers a place to express emotions immediately."* They also appreciated the flexible duration of the interactions. However, one challenge that participants faced was being able to find a private time to complete the interaction, as many of them shared a bedroom with a roommate.

As in the worksheet and chatbot conditions, several participants in the robot condition appreciated that they could talk to Blossom without the fear of being judged that comes with interacting with a human. P7 stated that the robot *"made me feel like I could talk about things without judgement. I think it's nice to know that the view that the AI has will be mostly objective."* P3 recalled that, at first, they *"didn't want to talk about anything too personal [...] But later [...] I knew it wouldn't judge me [...] I think people are wired to*

have some biases but this is a robot without emotions that doesn't really think or judge." P10 commented on the accessibility this technology could bring to mental health support, suggesting that Blossom could *"encourage [people] to get therapies in ways they otherwise might not, especially if they're introverted or antisocial. It would be good for them."* A few participants recalled bringing their issues to Blossom when their friends didn't have time to talk without having to worry about wasting Blossom's time. However, upon reflecting about discussing their issues with Blossom, one participant expressed shame about having to talk to a robot because they didn't have any close friends to confide in.

3.2 General Comments

Increased Guidance

A few participants across the three conditions wished they had received more guidance on how to complete the exercises. Participants across all three conditions expressed wanting more variety in the exercises provided, instead of having to choose between two. P17 (chatbot) stated *"Only having the 2 exercises was kind of limiting; there's only so much you can do with coping mechanisms and cognitive restructuring."* P32 (worksheet) suggested *"doing more than 2 options for the exercise [...] the two were great but [...] [the activity would be better] if there was more variety."* They shared that completing these exercises became repetitive by the end of the study. This feedback was especially prevalent in the worksheet condition, where the participants expressed frustration with filling out the same questions every day.

Interaction Design

Participants in all conditions expressed that the website was easy to navigate and did not have any issues. Some shared feedback about the interaction design.

One participant in the robot condition expressed wishing that they could verbalize their responses instead of typing them out, while another participant appreciated how typing encouraged reflection. Participants in the robot and chatbot conditions wished that they could practice the suggested coping strategies during the interaction. P6 (robot) suggested *"When [the agent] gives a list of potential ways to practice this thought pattern or solution, it might be helpful to offer to practice it."* A few participants in the chatbot condition observed that two weeks may not be enough time to observe changes in mental health or behavior. They had mixed thoughts on the duration of the interaction: some reported that a 30-minute interaction was too long and interrupted their day, while others thought the duration was ok and appreciated having control and flexibility in the duration of their interaction with the chatbot. Participants across all conditions expressed that they appreciated the daily reminders to complete the exercises.

Pre- and Post-Surveys

Some participants across conditions found that completing the pre- to post-session surveys was helpful for self-reflection. They shared that they were able to recall their previous answers and could use it as a measure of how much their feelings had improved as a result of the interaction. *"It is good to check in on thoughts, pre and post surveys were helpful"* (P14, robot); *"The survey questions enhanced my ability to dig up feelings"* (P32, worksheet). However, a few participants shared that they found the surveys to be too long and that they disincentivized them from completing the interaction. P24 explained, *"I wanted to talk to [the chatbot] once in the second week, but it would be annoying to fill out the surveys, so it was not worth the effort."*

Participant Comfort over Time and Privacy Concerns

Across all three conditions participants shared concerns for their privacy, wanting *"better knowledge of what [the researchers] are doing with the surveys and data"* (P31, worksheet). A few participants in the robot condition shared that because of these concerns they were less vulnerable in their discussions. A theme that emerged across all the conditions was that over time, participants became more comfortable writing their thoughts. Participants in the chatbot condition shared that they opened up more over time. This sentiment was most prevalent in the robot condition. Participants shared that while they were initially hesitant, they became more comfortable and open over time. One participant said they felt comfortable knowing what to expect from the system, and several others shared that becoming familiar with the system allowed for deeper reflection.

Participants in the chatbot and robot conditions shared that they were initially unsure what to expect, with some coming into the study with low expectations: *"I didn't really have a way to navigate what to say. I was definitely holding back [...] It was definitely a fear of the unknown for sure."* (P28, chatbot). However, they shared that both systems exceeded their expectations. Some participants in the chatbot and robot conditions shared that it took time to learn to interact with the chatbot, and several others shared that becoming familiar with the system allowed for deeper reflection. P6 (robot) recalled *""The interactions became a lot more organic for me [. . .] Over time it got a lot more casual. I got less strict with myself [...] realizing I really can ask anything I want to and it would be fine"*. Participants in all three conditions reported becoming more open and comfortable disclosing personal thoughts to their respective platforms over time. P4 (robot) stated *"As I was moving through [the] 7 days, I was opening up a little more each day."* P28 (chatbot) recalled, *"I struggle with body positivity in a lot of areas. I didn't share that with the chatbot [at first] but then [I] started to share it."* P7 (robot) shared that the topics of discussion became more intimate over time, *"It was easier for me to talk to the robot on day 8 rather than day 1. [At first] I didn't know what to tackle or talk about. I just talked about surface-level stresses like financial aid or family interaction as opposed to later [on, I talked about] about my struggles in isolation and connection."*
